# Surgery

**Published:** 1906-03-17

**Authors:** 


### SURGERY.

(*Continued from page 391.*)

*The Treatment of Intracranial Hæmorrhages.*—The diagnosis and treatment of a ruptured middle meningeal artery is not such a simple affair as would be gathered from text-books. Pringle[12] discusses the whole subject in relating 15 cases of his own. The commonest cause of this condition is fracture of the skull, but many cases are recorded in which the artery was severed and the skull uninjured. Also a fracture may occur on one side of the skull and a ruptured artery by contre-coup on the opposite. And one artery may be directly torn by the fracture, and the opposite ruptured by contre-coup. The clot may be situated in the temporal or fronto-temporal or parieto-temporal region, but in one case it was at the base of the brain in the sphenoidal region. The classical symptom of a lucid interval intervening between concussion and compression was seen in only a small proportion of the cases. In one case, which seems to be unique, consciousness was never lost throughout until four hours before death. But in this case copious loss of blood and cerebro-spinal fluid from the ear probably prevented a condition of cerebral compression. In nine out of the 15 cases no paralysis was present. But the presence of a definite hæmatoma in the temporal region is of great importance in diagnosis and treatment, because in many cases its exploration leads to the discovery of a fractured skull and an extradural hæmorrhage. The result of treatment is very disappointing. Of the 15 cases 10 were operated upon, and only four recovered. The explanation of this is probably twofold. First, that the brain is injured fatally at the same time that the artery is ruptured, and that if the clot be not evacuated at once the brain has not the power to re-expand, and so the compression remains unrelieved. It would seem a desperate thing to propose to operate for the intracranial hæmorrhages of the new-born, but Cushing[13] has written a paper which shows that this is by no means the case. Many children who are still-born, or who die of convulsions within a few days of birth, were found by the author to have extensive sub-dural hæmorrhages. Many of such cases, however, may survive, and after going through a latent period in which no symptoms are noticed, they develop the hopeless spastic palsy of one or more limbs with more or less complete amentia that forms the typical picture of a "birth palsy." That it is possible for such cases to survive at all is probably due to the distensibility of the fontanelles and to the fact that in early life the infant is almost entirely a bulbo-spinal animal in which the cerebrum has but little function. The hæmorrhages occur chiefly in the median region of the convexity of the brain. The actual vessels torn are the veins which run from the cerebral surface to the longitudinal sinus. As they pass from the pia mater to the dural sinus they are so unsupported as to be very liable to be injured. When the head is violently compressed either by the walls of the pelvis or by the forceps the edges of the parietals overlap, and this will easily cause rupture of the aforementioned veins. The diagnosis of a subdural hæmorrhage in an infant is not always difficult. In the first place, the condition of the fontanelle gives a clue which can never be obtained in the adult. A bulging fontanelle which does not pulsate is the most valuable sign. Convulsions, which may be unilateral, and ocular palsies are conspicuous, but with the exception of that affecting the eyes no motor paralysis is seen. A lumbar puncture will show blood corpuscles in the cerebro-spinal fluid. As the child grows up, amentia, spastic paraplegia, or diplegia, or epilepsy appear, and then nothing can be done by way of treatment. In four such cases Cushing has operated within the first few days of birth. One case died on the table, one died the next day, but the other two cases made good recoveries, and have every prospect of escaping the after-paralysis. A skin incision is made round the margins of the parietal bone, and the skin and bone turned down after cutting round the bone with scissors. The dark plum-coloured dura bulges into the wound, and is also turned down as a flap. The clot is removed and the two layers of dura and cranium stitched up without drainage. In one of the successful cases this operation was performed on both sides at the same sitting, and though the child had had many convulsions (six a day) and had great exophthalmos of one eye, it completely recovered and had no more fits.

**The Surgery of the Peripheral Nerves.**—It is only recently that many diseases of the peripheral nerves have come into the domain of surgery. This is notably the case with *brachial birth palsies*, when in many instances an irreparable and life-long damage is done to the brachial plexus during birth, and produces a most serious paralysis and deformity of the arm. Clark, Taylor, and Prout [14] have made a careful clinical and pathological study of this condition, based upon experiments on 25 infant cadavers and upon seven cases which were operated upon. It is shown that the condition always results from a traction force which pulls the head and shoulders apart. And that when this force is exerted so as to tear the brachial plexus, the nerves give way in order from above downwards. This explains the fact that the fifth and sixth cervical nerves are those most often injured. It would seem strange that regenerative processes do not take place more often, seeing that the ends of the divided nerves must remain in close contact. But by careful sections of portions of the plexus excised from the damaged areas, it is proved that the first event in the torn nerve trunk is the formation of a hæmatoma by a rupture of the vascular perineurium. The hæmatoma is replaced by fibrous tissue whose contraction compresses and destroys any growing nerve filaments. Hence it will be necessary in such cases to excise the cicatrix. The symptoms of brachial birth palsy are those of paralysis and atrophy of the muscles supplied by the injured nerves. In its most common form the paralysis affects the deltoid, the small rotators of the humerus, the biceps and supinator muscles, so that the arm lies at the side of the trunk, over-pronated and rotated inwards by the pectoralis major. As regards subjective symptoms, an important distinction has to be made between cases with and without pain. In the former a neuritis probably exists which will be made worse by massage and electricity. In the latter it will be necessary to employ these remedies long and thoroughly. If, however, at the end of a year no sign of improvement is seen in the paralysed muscles, then an operation ought to be undertaken. If only the nerves forming the upper trunk of the plexus are involved—*i.e.*, the fifth and sixth cervical—an incision across the posterior triangle will suffice to expose the field of operation. If the lower trunks are torn as well, then the clavicle and subclavius must be divided. The damaged areas in the plexus can be seen and felt as hard masses of scar tissue. They are excised by a sharp scalpel and the nerve ends carefully sewn together and wrapped round with Cargile membrane. Of the seven cases reported two died as the result of the operation, and of the remainder all showed a great improvement, both in muscular growth and power of voluntary movement. The high mortality could possibly be much lessened by adopting Crile's method of injecting cocaine or its modifications into the nerves before division, so as to lessen shock.

The *treatment of trigeminal neuralgia* is another subject in which surgery has only recently become ascendant. The excision of the Gasserian ganglion in the immediate relief of pain and its prospect of permanent cure is nevertheless a very formidable proceeding, and one which involves a risk of from 5 to 20 per cent. mortality. And Delbet [15] advocates the excision of the superior cervical sympathetic ganglion as the best method of treatment if it be adopted before the case has become too advanced. This proceeding, which was first practised by Jaboulay, is founded on the fact that the fifth nerve has many and intimate connections with the sympathetic. It has been proved by clinical and experimental evidence that resection of the superior cervical ganglion results in degeneration of the filaments which it gives off, and also in modifications in the cord and bulb at the level of the origin of the fifth nerves. There have been 20 cases recorded of this operation for the relief of facial neuralgia. It appears to give results which are intermediate between those resulting from excision of the Gasserian ganglion and those from division of peripheral branches of the nerve. That is to say, in the majority of cases great or almost complete relief from pain is afforded. And this relief extends over a long period (the longest mentioned being two years and ten months). But against the uncertainty of the method must be placed its freedom from risk and the absence of unsightly scars. The results are much better in comparatively recent cases. It seems, therefore, that if these observations are correct, that sympathicectomy will be the best treatment to adopt early in a case of trigeminal neuralgia and Gasserian excision can be adopted later on as a *dernier résort*.

[12] Scottish Med. and Sur. Jour., Feb. 1906. [13] Amer. Jour. of Med. Sc., Oct. 1905. [14] Ibid. [15] Brit. Med. Jour. Epit., Oct. 28, 1905.

